# Impact of antimicrobial stewardship fee on prescribing for Japanese pediatric patients with upper respiratory infections

**DOI:** 10.1186/s12913-020-05288-1

**Published:** 2020-05-11

**Authors:** Yuichi Muraki, Yoshiki Kusama, Masaki Tanabe, Kayoko Hayakawa, Yoshiaki Gu, Masahiro Ishikane, Daisuke Yamasaki, Tetsuya Yagi, Norio Ohmagari

**Affiliations:** 1 Department of Clinical Pharmacoepidemiology, Kyoto Pharmaceutical University, 5, Misasagi-Nakauchi-cho, Yamashina-ku, Kyoto-shi, Kyoto, 607-8414 Japan; 2 Antimicrobial Resistance Clinical Reference Center, Disease Control and Prevention Center, National Center for Global Health and Medicine, Tokyo, Japan; 3 Department of Infection Control and Prevention, Mie University Hospital, Mie, Japan; 4 Department of Infectious Diseases, Nagoya University Graduate School of Medicine, Nagoya, Japan

**Keywords:** Antimicrobial stewardship, Fees and charges, Prescriptions, Antimicrobial resistance, Upper respiratory tract infections

## Abstract

**Background:**

In 2018, the Japanese medical reimbursement system was revised to introduce a fee for the implementation of an antimicrobial stewardship (AS) fee for pediatric patients. The purpose of this study was to evaluate physicians’ prescription behavior following this revision.

**Methods:**

We conducted a retrospective observational study from January 1, 2017 to September 30, 2018 of pediatric (< 15 years) outpatients with upper respiratory tract infections (URIs). To assess the pattern of antibiotic prescription for the treatment of pediatric URIs before and after the introduction of the AS fee, we extracted data on pediatric URIs, diagnosed during the study period. Patients were divided based on whether medical facilities claimed AS fees. We defined antibiotic use as the number of antibiotics prescribed, and evaluated the proportion of each class to the total number of antibiotics prescribed. We also recorded the number of medical facilities that each patient visited during the study period.

**Results:**

The frequency of antibiotic prescription decreased after AS fee implementation, regardless of whether the facility claimed the AS fee, but tended to be lower in facilities that claimed the fee. Additionally, the frequency of antibiotic prescription decreased in all age groups. Despite the reduced frequency of antibiotic prescription, consultation behavior did not change.

**Conclusions:**

The AS fee system, which compensates physicians for limiting antibiotic prescriptions, helped to reduce unnecessary antibiotic prescription and is thus a potentially effective measure against antimicrobial resistance.

## Background

Antimicrobial resistance (AMR) is a growing global threat because in addition to its appreciable clinical impact, it also has a considerable economic impact [1, 2]. Therefore, it is necessary to reduce the prescription of inappropriate antibiotics and to control the spread of resistant bacteria not only in developing countries, but also in developed countries [2, 3]. Accordingly, the Japanese government developed the National Action Plan on Antimicrobial Resistance 2016–2020 in 2016 [4]. A prominent feature of the national action plan was the specification of outcome measures, including a reduction of antibiotic consumption and a reduction of the resistance rate. To achieve these outcomes, each health facility in Japan has implemented an antimicrobial stewardship (AS) program which aimed to stop the use of unnecessary antibiotics.

A common setting for antibiotic overuse is in the treatment of upper respiratory tract infections (URIs). The majority of URIs are viral [5], and the administration of antibiotics in the treatment of these conditions, or in preventing complications, has limited effectiveness [6]. In Japan, antibiotics were prescribed in 60% of clinical visits due to URIs, with third-generation cephalosporins, macrolides, and fluoroquinolones being among the most commonly prescribed antibiotics [7].

A high percentage (> 94%) of antimicrobials prescribed are oral antibiotics in Japan [8]. The use of oral antibiotics is reportedly higher in children (< 15 years) and the elderly (≥65 years) than in individuals aged 15–64 years [9]. Additionally, the highest prescription rates of all antimicrobials is among children aged 1–5 years, peaking at age 1 year [10]. Efforts to improve antibiotic prescribing practices need to incorporate some complementary strategies: changing physicians’ behavior [11], patient education, and modifying the healthcare system [12, 13].

In response to the current situation in Japan, the Japanese government developed the Manual of Antimicrobial Stewardship based on the national action plan on AMR in 2017 [14], and as a completely new and unique approach revised the Japanese medical reimbursement system in 2018, introducing a fee for the implementation of AS for pediatric patients with URIs of 800 Yen (approximately $7.5 US) for the first visit [15]. Pediatricians can claim the AS fee provided that they (1) have examined the patient for acute URI or acute diarrhea, with no other underlying condition, and (2) provide proof that they have provided education to the responsible adult on the condition for which they were consulted, including the lack of necessity for antibiotics.

In Japan, citizens have unrestricted access to medical care due to the public insurance system. A recent survey reported that 30.1% of patients want to be prescribed antibiotics for a common cold [16]. The implementation of the AS fee raised concerns that patients may seek care elsewhere if they were not prescribed antibiotics. Therefore, the purpose of this study was to evaluate the physician’s prescription and the patient’s behavior with respect to the management of pediatric patients with URIs following the implementation of the AS fee using claims data.

## Methods

### Study design

We conducted a retrospective observational study from January 1, 2017 to September 30, 2018. Data were obtained from the claims database, IQVIA Claims, which collects data of corporate employees and their family members. This database consists of anonymized inpatient, outpatient and pharmacy claims data on > 2.3 million individuals aged < 75 years from 32 Society-Managed Health Insurance institutions. The data accounts for approximately 5% of the total number of claims. In addition, the database includes patient characteristics (sex, and 5-year age group), prescription drug information (brand name, generic name, prescription date, duration), diagnostic information (date of the initial diagnosis and treatment days), treatment procedure (operation, radiation, examination, etc.), and hospital characteristics (classified according to the number of beds and specialty).

The study was performed in accordance with the Declaration of Helsinki and the study protocol was approved by the Ethics Committee of Kyoto Pharmaceutical University (No. 19–13). As this study used only anonymized claims data, the requirement for the consent was waived according to the requirements of the Japanese Ethical Guidelines for Medical and Health Research Involving Human Subjects [17].

### Patients

**Fig. 1:**
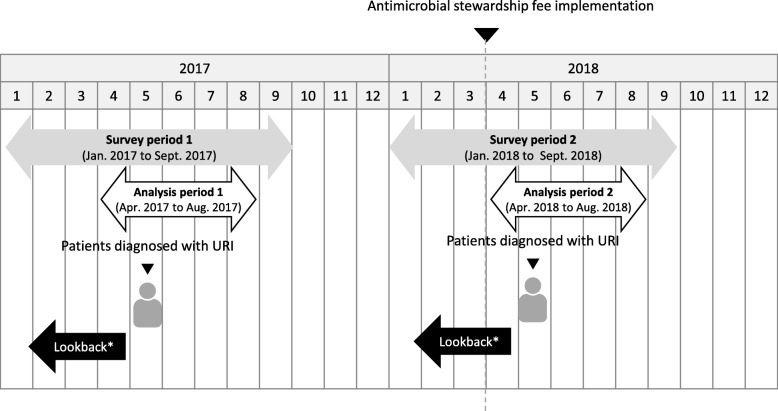
Timeframe for data extraction. Patients who met all the following conditions were enrolled in this study: (1) diagnosed with an upper respiratory tract infection (URI) during the survey period; (2) aged < 15 years in the month of diagnosis; (3) pediatrician claimed an initial visit fee in the month of diagnosis; (4) diagnosed with a different condition within 3 months prior to the diagnosis of the URI and not hospitalized (Lookback*).URI: upper respiratory tract infection

We evaluated pediatric patients aged < 15 years who visited outpatient clinics for treatment of an acute URI or acute tonsillitis, as identified by their respective Japanese disease codes (4,659,007 and 8,832,289). To assess the antibiotic prescription pattern for the treatment of URIs before and after the AS fee was implemented, we extracted data for pediatric URIs diagnosed during the study period, and divided them according to whether the medical facility had claimed the AS fee (Fig. 1). Children who met all the following conditions were included in the study: (1) diagnosed with URIs in the survey period; (2) aged < 15 years in the month of diagnosis; (3) pediatrician claimed an initial visit fee in the month of diagnosis; (4) had been diagnosed with another condition within the 3 months prior to the URI diagnosis and had not been hospitalized. Additionally, we evaluated the impact of the AS fee on antibiotic prescription according to age group.

### Data collection

Antibiotics were classified according to Level 4 of the Anatomical Therapeutic Chemical Classification System. We classified antibiotic use according to the number of antibiotics prescribed and evaluated the proportion of each class in relation to the total number of antibiotics prescribed. We also determined whether the same patient visited multiple facilities during the study period.

### Statistical analysis

Data analysis was performed using JMP Pro 14 (SAS Institute Inc., Cary, NC, USA) and IBM SPSS Statistics 23 (IBM, Armonk, NY, USA). Most of the results were reported as frequencies and proportions. Pearson’s chi-square test was used to test for statistical significance of the differences in the proportion of children prescribed an antibiotic before and after the introduction of the AS fee. Statistical significance was defined as a *p*-value < 0.05.

## Results

**Fig. 2:**
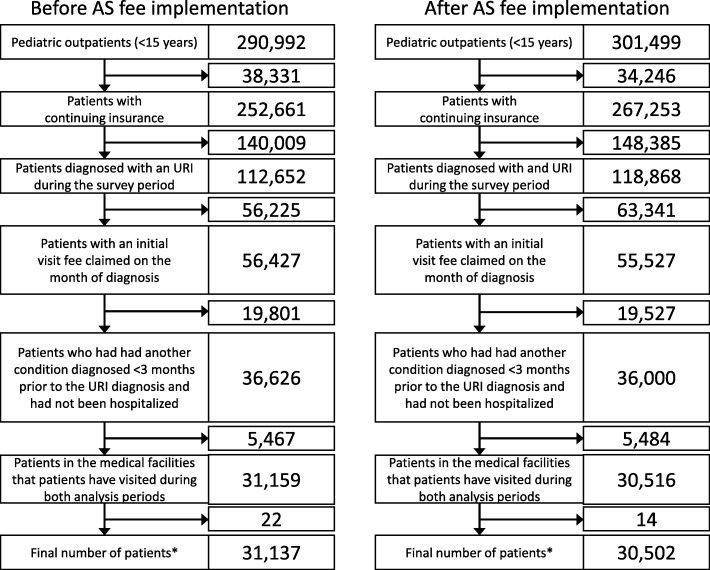
Flowchart showing patient selection and the number of exclusions due to ineligibility. * We excluded multiple visits during the analysis period. AS: antimicrobial stewardship

Figure 2 shows the process for selecting children for inclusion in the analysis, and the number of children who were excluded because they did not meet the inclusion criteria. There were 31,137 children who met the criteria for inclusion in the “Before” group, and 30,502 children who met the criteria for inclusion in the “After” group.

**Fig. 3:**
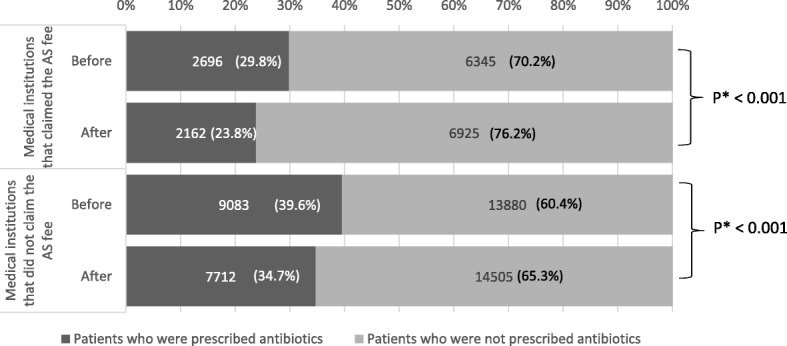
Impact of the antimicrobial stewardship fee on the rate of antibiotic prescription. We divided the number of patients visiting a hospital by the rate of antibiotic prescription to calculate the percentages in the graph. *Pearson’s chi-square test, AS: antimicrobial stewardship

The frequency of antibiotic prescription for URIs decreased significantly after the AS fee implementation, regardless of whether the facility claimed the fee (Fig. 3).

The frequency of antibiotic prescription was lower in facilities that claimed the AS fee than in facilities that did not both before and after AS fee implementation. Additionally, the magnitude of the decrease was similar in facilities that claimed the AS fee (6.03, 95% confidence interval [CI]: 4.74–7.32%) and those that did not claim the AS fee (4.84, 95% CI: 3.95%–5.73), but there was no marked change in the overall antibiotic prescribing pattern (Table 1).

**Table 1:** Antibiotic prescription frequency before and after the implementation of the antimicrobial stewardship fee^a^

	Medical facilities that claimed the AS fee^b^		Medical facilities that did not claim the AS fee^b^		All medical facilities^b^	
	Before	After	Before	After	Before	After
Tetracyclines (J01AA)	15 (0.5)	6 (0.3)	40 (0.4)	26 (0.3)	55 (0.4)	32 (0.3)
Penicillins (J01CA, CE)	551 (19.3)	540 (23.7)	1559 (16.0)	1572 (19.0)	2110 (16.8)	2112 (20.0)
Combinations of penicillins, including beta-lactamase inhibitors (J01CR)	37 (1.3)	35 (1.5)	269 (2.8)	246 (3.0)	306 (2.4)	281 (2.7)
First-generation cephalosporins (J01DB)	27 (0.9)	13 (0.6)	44 (0.5)	46 (0.6)	71 (0.6)	59 (0.6)
Second-generation cephalosporins (J01DC)	29 (1.0)	27 (1.2)	82 (0.8)	82 (1.0)	111 (0.9)	109 (1.0)
Third-generation cephalosporins (J01DD)	1426 (50.1)	1032 (45.2)	4714 (48.4)	3776 (45.7)	6140 (48.8)	4808 (45.6)
Other beta-lactams (J01DE, DF, DH)	39 (1.4)	26 (1.1)	180 (1.1)	147 (1.8)	219 (1.7)	173 (1.6)
Combinations of sulfonamides and trimethoprim, incl. Derivatives (J01EE)	0 (0.0)	0 (0.0)	2 (0.0)	1 (0.0)	2 (0.0)	1 (0.0)
Macrolides (J01FA10)	567 (19.9)	472 (20.7)	2272 (23.4)	1880 (22.7)	2839 (22.6)	2352 (22.3)
Fluoroquinolones (J01MA)	117 (4.1)	100 (4.4)	457 (4.7)	385 (4.7)	574 (4.6)	485 (4.6)
Aminoglycosides (J01GA, GB)	0 (0.0)	0 (0.0)	0 (0.0)	0 (0.0)	0 (0.0)	0 (0.0)
Glycopeptide antibacterials (J01XA)	0 (0.0)	0 (0.0)	0 (0.0)	0 (0.0)	0 (0.0)	0 (0.0)
Other antibacterials (J01XB-XX)	41 (1.4)	32 (1.4)	111 (1.1)	106 (1.3)	152 (1.2)	138 (1.3)
Total	2849	2283	9730	8267	12,579	10,550

^a^The values are based on the information from the participating facilities claiming the antimicrobial stewardship fee or not

^b^The values show the total frequency of prescription (proportion)

Abbreviations: *AS* antimicrobial stewardship

**Fig. 4:**
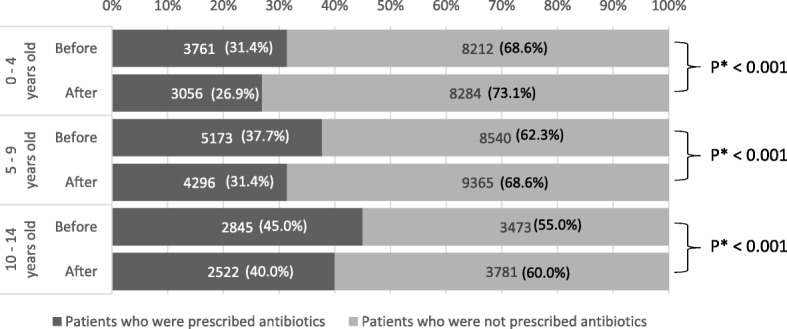
Antibiotic prescription frequency before and after antimicrobial stewardship fee implementation, according to age. The number of patients visiting a hospital was divided by the rate of antibiotic prescription to calculate the percentages in the graph. * Pearson’s chi-square test

The frequency of antibiotic prescription for URIs decreased significantly in all age groups after AS fee implementation (*p* < 0.001, Fig. 4). Despite the reduced frequency of antibiotic prescription, consultation behavior did not change, and the majority of children had only one medical facility visit before (97.3%) and after (97.4%) implementation of the AS fee (*p* = 0.06).

## Discussion

After the implementation of the AS fee, the frequency of prescriptions for children with URIs decreased at both facilities that did, and did not claim the AS fee, and the frequency of antibiotics decreased in all age groups. However, there was no marked change in either the frequency of consultations, or in the overall prescribing pattern.

In this study, the frequency of prescriptions for children with URIs decreased regardless of the type of facility. It has been reported that educational interventions, awareness-raising activities, feedback on social norms, and restrictions have reduced the frequency of prescriptions [11, 13, 18]. In Japan, the AMR Clinical Reference Center (AMRCRC) was established in 2017 as a commissioned project by the Ministry of Health, Labor and Welfare. The AMRCRC is mainly overseeing the awareness activities for AMR toward public and medical professionals, the construction of surveillance systems, and the release of epidemiological data [19, 20]. Therefore, it has been suggested that these activities may influence prescription frequency regardless of whether there is an AS fee claim. On the other hand, the finding that the frequency of prescriptions tends to be less in medical facilities that claimed the AS fee, suggests that having a system that rewards medical facilities that take appropriate action is an important measure.

The use of antibiotics in children peaks at the age of one year in Japan [10]. In other countries antibiotics also tend to be prescribed more frequently in younger children [21]. Notably, this intervention reduced prescriptions among patients of all ages. This suggests that the AS program may change physicians’ overall prescribing behavior.

In October 2017, the estimated number of patients with acute URIs aged < 15 years was 113,500 per day, and the number of pediatric clinics was 19,647 [22]. One clinic reported that the AS fee increased the average amount spent per patient from 5490 Yen in FY 2017, to 6300 Yen in FY 2018 (unpublished data), so the cost related to the AS fee might be low as a proportion of the total medical fee. There have been reports on the giving of incentives to hospitals, general practitioners, and county units for the reduction of antibiotic prescriptions in England [23, 24] and Sweden [25]. However, our approach to providing incentives for physicians’ behavior, to not only reduce antibiotic prescribing but also to provide education to patients is, to our knowledge, unique to Japan. Although mid- to long-term evaluation is necessary, the approach used in our study might also be effective in reducing inappropriate antibiotic use in other countries.

Our study did not show an increase in multiple consultations after the implementation of the AS fee. The AS fee provides doctors with an incentive not to prescribe antibiotics at the first visit and rewards them when they manage and educate patients without prescribing antibiotics [15]. It is assumed that children’s parents will be satisfied with the explanations that the doctor has provided.

This study has some limitations. Firstly, it is not an evaluation of the entire population because it only targeted children and used the claims data from 32 Society-Managed Health Insurance institutions responsible for about 5% of claims; thus, the generalizability of the findings might be limited. However, it provides valuable information for evaluating the effectiveness of the AS fee using claims data. A national database could be used in future studies to broaden the generalizability of the findings. Another limitation is that individual patient outcomes could not be evaluated. The results might have also been influenced by the physician not prescribing antibiotics on the first visit. Additionally, the analysis period was less than a year because the claims database could not yet be obtained when we conducted this study. Our research provides vital information that can be a basis for implementing AS fees in other countries. This methodology that evaluated using claims data might also be valuable in other medical reimbursement systems.

## Conclusion

In conclusion, although the frequency of antibiotic prescriptions decreased regardless of whether the facility claimed the AS fee, the AS fee helps to limit unnecessary antibiotic prescription without affecting patient behavior. Thus, the AS fee system can be considered an effective strategy against AMR.

## Data Availability

The data that support the findings of this study are available from IQVIA Solutions Japan K.K. but restrictions apply to the availability of these data, which were used under license for the current study, and so are not publicly available. Data are however available from the authors upon reasonable request and with permission of IQVIA Solutions Japan K.K.

## Abbreviations

AMR: Antimicrobial resistance
AS: Antimicrobial stewardship
URI: Upper respiratory infection
FY: Fiscal year
AMRCRC: AMR Clinical Reference Center

## References

[1] World Health Organization. Global action plan on antimicrobial resistance: World Health Organization; 2015. Available from http://www.who.int/iris/handle/10665/193736. Accessed 8 March 2019. 10.7196/samj.9644 26242647

[2] Hofer U (2019). The cost of antimicrobial resistance. Nat Rev Microbiol.

[3] Founou RC, Founou LL, Essack SY (2017). Clinical and economic impact of antibiotic resistance in developing countries: A systematic review and meta-analysis. PLoS One.

[4] The Government of Japan. National action plan on antimicrobial resistance (AMR) 2016–2020. Available from: https://www.mhlw.go.jp/file/06-Seisakujouhou-10900000-Kenkoukyoku/0000138942.pdf. Accessed 8 March 2019.

[5] Kronman MP, Zhou C, Mangione-Smith R (2014). Bacterial prevalence and antimicrobial prescribing trends for acute respiratory tract infections. Pediatrics..

[6] Meropol SB, Localio AR, Metlay JP (2013). Risks and benefits associated with antibiotic use for acute respiratory infections: a cohort study. Ann Fam Med.

[7] Hashimoto H, Matsui H, Sasabuchi Y, Yasunaga H, Kotani K, Nagai R (2019). Antibiotic prescription among outpatients in a prefecture of Japan, 2012-2013: a retrospective claims database study. BMJ Open.

[8] Muraki Y, Yagi T, Tsuji Y, Nishimura N, Tanabe M, Niwa T (2016). Japanese antimicrobial consumption surveillance: first report on oral and parenteral antimicrobial consumption in Japan (2009–2013). J Glob Antimicrob Resist.

[9] Yamasaki D, Tanabe M, Muraki Y, Kato G, Ohmagari N, Yagi T (2018). The first report of Japanese antimicrobial use measured by national database based on health insurance claims data (2011-2013): comparison with sales data, and trend analysis stratified by antimicrobial category and age group. Infection..

[10] Kinoshita N, Morisaki N, Uda K, Kasai M, Horikoshi Y, Miyairi I (2019). Nationwide study of outpatient oral antimicrobial utilization patterns for children in Japan (2013-2016). J Infect Chemother.

[11] van der Velden AW, Kuyvenhoven MM, Verheij TJ (2016). Improving antibiotic prescribing quality by an intervention embedded in the primary care practice accreditation: the ARTI4 randomized trial. J Antimicrob Chemother.

[12] Wei X, Zhang Z, Hicks JP, Walley JD, King R, Newell JN (2019). Long-term outcomes of an educational intervention to reduce antibiotic prescribing for childhood upper respiratory tract infections in rural China: follow-up of a cluster-randomised controlled trial. PLoS Med.

[13] Jacobs TG, Robertson J, van den Ham HA, Iwamoto K, Bak Pedersen H, Mantel-Teeuwisse AK (2019). Assessing the impact of law enforcement to reduce over-the-counter (OTC) sales of antibiotics in low- and middle-income countries; a systematic literature review. BMC Health Serv Res.

[14] Infectious Diseases Control Division (2017). Health Service Bureau, Ministry of Health, Labour and Welfare. Manual of Antimicrobial Stewardship.

[15] Ministry of Health, Labour and Welfare, Japan. The fiscal year (2018). Revision of Reimbursement of Medical Fees.

[16] AMR Clinical Reference Center, Japan (2018). Antibiotic awareness survey.

[17] Ministry of Health, Labour and Welfare (2014). Ethical guidelines for medical and health research involving human Subjects.

[18] Hallsworth M, Chadborn T, Sallis A, Sanders M, Berry D, Greaves F, et al. Provision of social norm feedback to high prescribers of antibiotics in general practice: a pragmatic national randomised controlled trial. Lancet. 2016;387:1743–1752. doi: 10.1016/S0140-6736(16)00215-4. 10.1016/S0140-6736(16)00215-4 PMC4842844 26898856

[19] AMR Clinical Reference Center, Japan. Japan Surveillance for Infection Prevention and Healthcare Epidemiology: J-SIPHE Available from https://j-siphe.ncgm.go.jp/home. Accessed 8 March 2019.

[20] AMR Clinical Reference Center, Japan. Surveillance of antimicrobial use Available from http://amrcrc.ncgm.go.jp/surveillance/010/20181128172333.html. Accessed 8 March 2019.

[21] Cassini A, Högberg LD, Plachouras D, Quattrocchi A, Hoxha A, Simonsen GS (2019). Attributable deaths and disability-adjusted life-years caused by infections with antibiotic-resistant bacteria in the EU and the European economic area in 2015: a population-level modelling analysis. Lancet Infect Dis.

[22] Ministry of Health, Labor and Welfare. Patient survey. https://www.mhlw.go.jp/toukei/saikin/hw/kanja/17/dl/01.pdf. Accessed 16 December 2019.

[23] Islam J, Ashiru-Oredope D, Budd E, Howard P, Walker AS, Hopkins S (2018). A national quality incentive scheme to reduce antibiotic overuse in hospitals: evaluation of perceptions and impact. J Antimicrob Chemother.

[24] Wise J (2016). Hospitals and GPs are offered incentives to reduce antibiotic prescribing. BMJ..

[25] Mölstad S, Löfmark S, Carlin K, Erntell M, Aspevall O, Blad L (2017). Lessons learnt during 20 years of the Swedish strategic programme against antibiotic resistance. Bull World Health Organ.

